# Green Picosecond Laser Machining of Thermoset and Thermoplastic Carbon Fiber Reinforced Polymers

**DOI:** 10.3390/mi12020205

**Published:** 2021-02-17

**Authors:** Insung Choi, Su-Jin Lee, Dongsig Shin, Jeong Suh

**Affiliations:** Laser Industrial Technology Research Group, Korea Institute of Machinery and Materials (KIMM), 48, Mieumsandan 5-ro 41beon-gil, Busan 46744, Korea; leesj@kimm.re.kr (S.-J.L.); dsshin@kimm.re.kr (D.S.); jsuh@kimm.re.kr (J.S.)

**Keywords:** picosecond laser, micromachining, thermoset, thermoplastic, carbon fiber reinforced polymer

## Abstract

There has been an increase in demand for the development of lightweight and high-strength materials for applications in the transportation industry. Carbon fiber reinforced polymer (CFRP) is known as one of the most promising materials owing to its high strength-to-weight ratio. To apply CFRP in the automotive industry, various machining technologies have been reported because it is difficult to machine. Among these technologies, picosecond laser beam-induced machining has attracted great interest because it provides negligible heat transfer and can avoid tool wear. In this work, we conducted and compared machining of 2.15 mm-thick thermoset and 1.85 mm-thick thermoplastic CFRPs by using a green picosecond laser. The optimized experimental conditions for drilling with a diameter of 7 mm led to a small taper angle (average ~ 3.5°). The tensile strength of the laser-drilled specimens was evaluated, and the average value was 570 MPa. Our study indicates that green picosecond laser processing should be considered as a promising option for the machining of CFRP with a small taper angle.

## 1. Introduction

The automotive industry has been confronted with the need for the development of lightweight materials for weight reduction to satisfy fuel efficiency requirements [1,2,3]. In an effort to provide both lightweight and high strength qualities, carbon fiber reinforced polymer (CFRP) has attracted great attention due to its high strength-to-weight ratio [4,5]. To apply CFRP in automotive vehicles, many researchers have endeavored to develop CFRP/metal dissimilar joining technology [6,7,8]. Self-piercing rivets (SPRs), a type of mechanical fastener for joining sheet materials, are considered as a high potential solution for CFRP/metal joining [9,10,11]. Unlike conventional SPR specimens, the hole drilling of CFRP is required to avoid galvanic corrosion of the rivet interface between CFRP and metal [12,13]. In particular, pre-hole machining is regarded as essential for SPR application to high-tension steel plates (HTSP), for the purpose of enhancing the safety of motor vehicles. Recently, a study on pre-holed SPR was presented using mechanical drilling [14]. Although CFRP has superior mechanical properties, machining is very difficult by conventional mechanical machining and water jet machining, which cause serious damage with the pulling out of fibers, cracking, delamination, tool wear, inadequate surface quality, and wastewater handling [15,16]. In this respect, laser material processing has been investigated as an alternative method for CFRP machining.

A variety of studies have been reported that have investigated the laser beam-induced machining of CFRP composites. Various experimental parameters regarding laser machining systems, including laser wavelength, pulse duration, repetition rate, beam size, scan speed, beam overlap, and power density, have been systematically examined with simulation analyses [17,18,19,20,21,22,23,24,25,26,27,28]. A previous study has found that the green wavelength has advantages regarding control of ablation depth due to shorter absorption length and lower ablation threshold fluence for carbon, compared to the infrared wavelength [17]. A picosecond laser can be used to process materials with relatively less influence on the heat-affected zone (HAZ) than nanosecond and CW laser machining [22,28]. In addition, many researchers have made an effort to improve cutting quality and decrease processing time by studying how kerf width, ablation depth, taper angle, edge quality, HAZ, and thickness influence CFRP [23,24,25,26,27]. Multiple parallel pass and multi-ring processing methods have been introduced as effective machining strategies [23,24,29,30]. An increase in scan speed, causing a decrease in beam overlap, was shown to reduce ablation depth [23,24]. Although, many studies using laser processing for industrial applications of CFRP have been reported, most experimental works have focused on the machining of thermoset CFRP using infrared laser wavelength. For the environmental aspect of recycling, a systematic machining study of thermoplastic CFRP is also potentially important for CFRP reuse [5,31,32,33].

In this work, we report on an investigation of the ultrafast laser machining of thermoset and thermoplastic CFRP. For the machining of CFRP, a picosecond laser is preferable for using at a high power, compared to a femtosecond laser. In respect of the ablation efficiency, a longer pulse, such as a 10 ps laser, can be better than a 1 ps laser. Our study aimed to directly compare the experimental parameters of thermoplastic and thermoset CFRPs. Therefore, a 1 ps laser was utilized for this work because thermoplastic CFRP is very sensitive to thermal effects. The green laser wavelength (λ = 515 nm) and a galvanometer scanning system were employed to minimize the heat effect so that the material would retain its high strength. A variety of experimental parameters were optimized to completely cut 2.15 mm-thick thermoset and 1.85 mm-thick thermoplastic CFRPs. To verify the cross-section of the millimeter-scale thickness and taper angles of the specimens at once from top to bottom, a stereoscopic microscope with low-magnification was employed. Although a green picosecond laser was utilized to reduce the heat effect, the thermoplastic CFRP required a faster scan speed and a small dwell time to minimize the thermal damage in comparison to the thermoset CFRP. The experimental parameters obtained from line cutting were referred for hole drilling works with a diameter of 7 mm. The optimized process resulted in a small taper angle of between 3° and 3.5°. The tensile strength was further evaluated to characterize the mechanical property of the laser-drilled samples, and it was compared with that of mechanically drilled holes. Our experimental parametric study on drilling by using a green picosecond laser demonstrated that it is an effective machining method for CFRP with a small taper angle.

## 2. Materials and Methods

### 2.1. Laser Micromachining System and Materials

**Figure 1:**
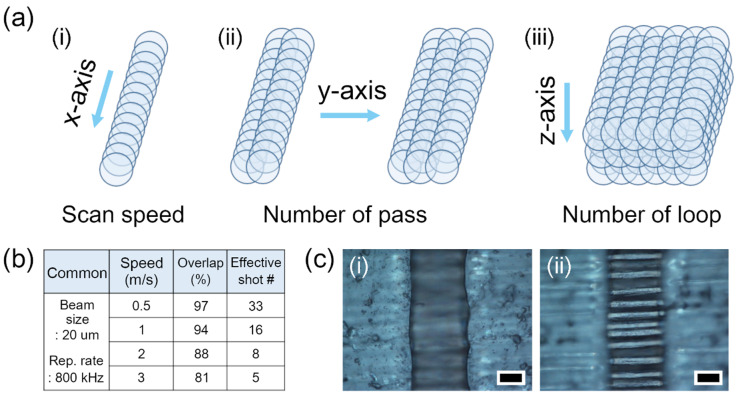
Laser machining using Gaussian spot beam and galvanometer scanner. (**a**) Schematic of (i) x, (ii) y, and (iii) z-axis scanning using laser spot beam. Main experimental parameters with a scan speed, the number of passes, and the number of loops corresponding to movement of each axis. One loop means a cycle with the number of passes in lateral direction. (**b**) Relationship between the beam size, the repetition rate, the scan speed, beam overlap, and effective shot numbers per area. (**c**) Optical microscope images of laser-machined carbon fiber reinforced polymer (CFRP) surface. The images indicate (i) top surface and (ii) carbon fiber bundles at a deeper focus position. Each scale bar = 20 μm.

The laser micromachining experiments were carried out by using a green picosecond laser (AMPHOS 400) with a galvanometer scanning system, and its specifications are given in Table 1. Figure 1a presents a schematic of the *x–y–z* axis machining that was applied to the specimens using a galvanometer scanner head (RAYLASE). To focus a laser spot beam onto a planar image plane, an f-theta focusing lens (JENOPTIK) was utilized with a focal length of 170 mm and telecentricity of 8.2°. In addition, a motion-controlled stage was employed to mount the CFRP specimens for initial positioning in the galvanometer scanning zone. The CFRP plates with plain weave structure (150 × 50 mm) were prepared with 2.15 mm-thick thermoset and 1.85 mm-thick thermoplastic composites. The binding polymers were epoxy and polycarbonate for the thermoset and thermoplastic, respectively. The thermal properties of the CFRP composites studied in this work referred to previous research articles [25,27,28,34].

### 2.2. Laser Micromachining Procedure

The main experimental parameters with a Gaussian spot beam are the scan speed (x-axis), the number of passes in the lateral direction (y-axis), and the number of loops, which has a direct influence on the machining depth (z-axis). The effective ablation area by laser beam overlap is determined by the scan speed, which ranged from 0.5 to 3 m/s in this study. For the laser beam-induced machining of thick materials, kerf width is an important parameter to reduce subsequent shielding of the laser beam by the plume generated. Thus, the number of passes in the lateral direction and the spacing between passes (here, called the gap) were systematically investigated for both thermoset and thermoplastic CFRPs. In addition, the laser power and dwell time were also investigated. To avoid thermal damage induced by heat accumulation, the laser powers were tested up to 37 W, and dwell times from 0.1 to 1.0 s were considered. Each power of 4.5, 9, 16, 22, 29, and 37 W corresponds to laser fluence (energy density) of 1.78, 3.58, 6.37, 8.76, 11.54, and 14.73 J/cm^2^, respectively. The common experimental conditions were a 515 nm wavelength, a pulse duration of 1 ps, and a repetition rate of 800 kHz. For potential applications in a manufacturing process, the experimental parameters used in this study were optimized in air. All the experiments for complete cutting and drilling were carried out at the same focus position (below 0.5 mm from the surface) without need for movement of the focal plane or positioning of the specimen in the z-axis. A long depth of focus (DOF) with 1.219 mm was utilized for this study.

At the beginning of this work, line cutting parameters were scrutinized to reduce the processing time and the effect on the heat-affected zone (HAZ). For the line cutting of the thermoset CFRP, it was possible to use higher power, and it was less sensitive to thermal effects in comparison to the thermoplastic CFRP. For both the thermoset and thermoplastic CFRPs, it was necessary to make a wider kerf width because of the small beam size (20 μm) of our laser system. To apply the picosecond laser machining method to the SPR for CFRP/metal dissimilar joining, a drilling study with diameter of 7 mm was performed based on multi-ring processing with reference to the line cutting parameters. A two-step process for the drilling of 2.15 mm-thick specimens was utilized to reduce the processing time and taper angle.

To observe the cross-section of the specimens at once from top to bottom (2.15 mm thickness), a stereoscopic microscope (Olympus SZ61) was utilized to obtain low-magnification images. The hole diameters on a millimeter-scale and taper angles were measured by using the same microscope. In addition, further inspections, such as examination of the HAZ area, were carried out by optical microscopy (Nikon MM-800). To compare the mechanical properties of specimens subjected to green picosecond laser drilling with those of specimens subjected to mechanical drilling, tensile strengths were examined using a universal testing system (Instron 5582). The test procedure conformed to KS M ISO 527-4.

## 3. Results and Discussion

Pulsed laser ablation is one of the most useful and precise methods of micromachining by the selective removal of materials [35,36]. The ablation of materials takes place above a certain threshold fluence (laser energy per unit area). The amount of threshold fluence not only depends on the optical and thermal properties of materials, but also on laser parameters, such as wavelength, pulse duration, and so forth. Above the ablation threshold, the volume of material removed per pulse shows a logarithmic rise by fluence increase according to the Beer–Lambert law [36]. In addition, the size of the focused laser beam and the heat penetration are also important parameters that affect the volume of laser-ablated material. The diameter of the beam size (2ω_0_) at focus can be obtained by

$$2\omega_{0}\;=\;\frac{4\lambda fM^{2}}{\pi d}$$

where ω_0_ is the beam waist radius, *λ* is the wavelength of the laser, *f* is the focal length, *M*^2^ is the beam quality factor, and d is the diameter of the entering beam. The ablated hole diameter *D* as a function of laser fluence ∅ is given by

$$D^{2}=2\omega_{0}{}^{2}\ln\left(\frac{\emptyset }{\emptyset_{th}}\right)$$

where $\emptyset_{th}$ is the ablation threshold. According to the above equation, the ablated hole diameter D is highly dependent on laser fluence. In this study, it is more or less difficult to evaluate the threshold fluence $\emptyset_{th}$ because the CFRPs have a heterostructure with a combination of polymer layers and carbon fiber bundles. With reference to a previous report by Wolynski et al. [17], we assumed a threshold fluence of 0.284 J/cm^2^ at the green wavelength and thereby the ablated hole diameter D can be calculated [36,37].

Figure 1a shows a schematic of *x*–*y*–*z* axis machining by laser beam scanning using a two-axis galvanometer scanner. The machining movement of each axis corresponds to the main experimental parameters of the scan speed, the number of passes, and the number of loops, respectively. The effective ablation area by laser beam overlap is determined by the beam spot size, the repetition rate of the laser, and the scan speed. For example, with a repetition rate of 800 kHz and a scan speed of 0.5 m/s, the laser beam moves 0.625 μm per pulse (Figure 1(ai)). This means that the beam movement is about 3.12% of the beam spot size (20 μm) and the beam overlap is about 97%. Here, we assumed a use of 2.1 J/cm^2^, for convenient calculation of the beam overlap, and thereby the beam spot size (D) is 20 μm according to the equation about diameter squared versus fluence method. From the relationship between the beam spot size (20 μm), the repetition rate (800 kHz), and scan speed (0.5 m/s), 33 pulses can be irradiated on the same area. Therefore, the scan speed is one of the important experimental parameters for machining, and its relationships with beam overlap and effective shot numbers (the number of pulse irradiations per area) are summarized in Figure 1b. The kerf width plays an important role in the cutting of thick materials. To obtain a proper lateral kerf width, the laser beam was overlapped on the y-axis with a gap of 10 μm in this study (Figure 1(aii)). After determination of the micromachining parameters for the x–y axis (here, assumed as one cycle), repeated processing of the cycle is required to cut the sample completely. For this reason, we regarded the repetitions of work on the x–y axis as the number of loops (Figure 1(aiii)). Figure 1c shows example optical microscope images focused on (i) the top surface and (ii) carbon fiber bundles of the thermoset CFRP after laser micromachining on the x-axis.

**Figure 2:**
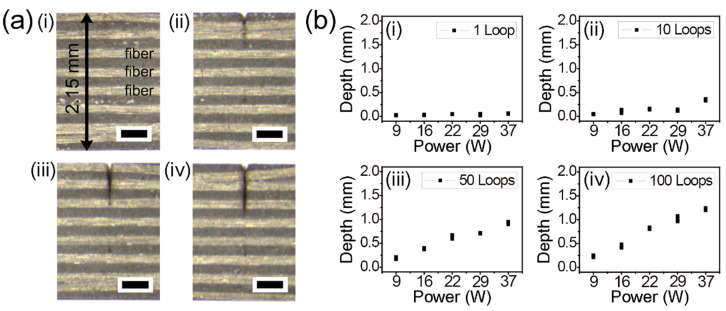
Picosecond laser cutting of thermoset CFRP with lateral single-pass processing. (**a**) Stereoscopic microscope cross-section images by a power of 22 W and (i) 1 loop, (ii) 10 loops, (iii) 50 loops, and (iv) 100 loops, respectively. Each scale bar = 400 μm. (**b**) Systematic investigation of cutting depths by power variation and the number of loops variation with (i) 1, (ii) 10, (iii) 50, and (iv) 100. The common experimental condition was a scan speed of 0.5 m/s.

A stereoscopic microscope with low magnification was utilized to clearly show a cross-section of the specimens at once from top to bottom (2.15 mm thickness). Figure 2 presents the experimental results of picosecond laser cutting of the thermoset CFRP using lateral single-pass processing. The images show cutting depths in relation to the number of loops, namely (i) 1, (ii) 10, (iii) 50, and (iv) 100 loops (Figure 2a). The common experimental conditions utilized in Figure 2a were a power of 22 W and a scan speed of 0.5 m/s. To verify the feasibility of cutting using single-pass processing, various experimental parameters were systematically investigated, as shown in Figure 2b. Even though power and the number of loops were increased up to 37 W and 100 loops, all the cases seemed to be unable to cut completely. In addition, we tried to move down the focal plane on the z-axis with reference to previous research articles [23,24]. However, complete cutting with single-pass processing required a very long processing time due to a narrow kerf width from the small beam size.

**Figure 3:**
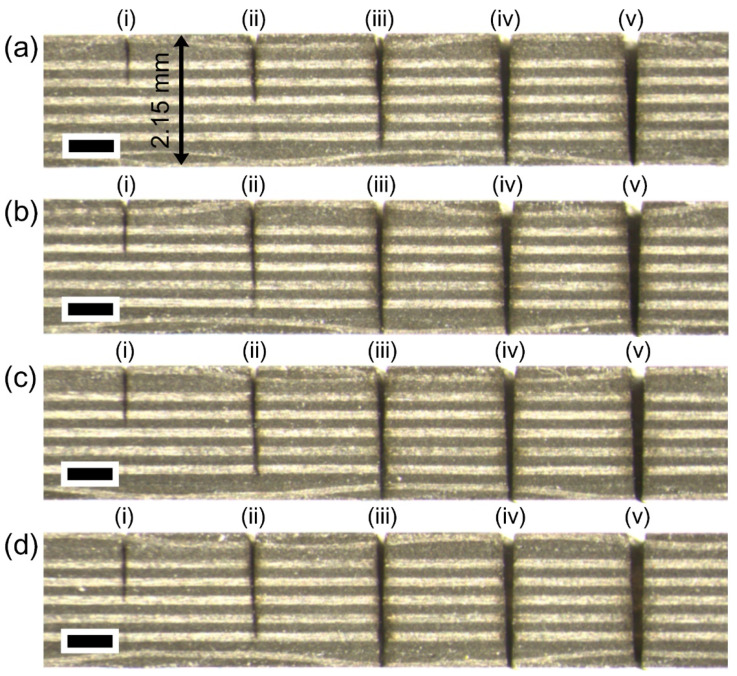
Picosecond laser cutting of thermoset CFRP with lateral multi-pass processing. Stereoscopic microscope cross-section images with (**a**) 50 loops, (**b**) 100 loops, (**c**) 200 loops, and (**d**) 300 loops, respectively. Each position at (i), (ii), (iii), (iv), and (v) corresponds to multi-pass processing of 1, 5, 10, 15, and 20 passes in lateral direction with 10 μm gap. The common experimental conditions were a power of 22 W and a scan speed of 0.5 m/s. Each scale bar = 700 μm.

Figure 3 demonstrates that with a proper kerf width, lateral multi-pass processing can cut CFRP completely. The effect of the number of passes was systematically investigated, specifically, 1, 5, 10, 15, and 20 passes in the lateral direction with a gap of 10 μm, which correspond to (i), (ii), (iii), (iv), and (v). The common experimental conditions in Figure 3 were a power of 22 W and a scan speed of 0.5 m/s. According to the equation regarding the diameter squared versus fluence method, a spot size is 26.2 µm has a fluence of 8.76 J/cm^2^ (800 kHz, 22 W). The stereoscopic microscope image in Figure 3a indicates that 50 loops are sufficient to completely cut the material using 15 passes. Furthermore, additional experiments with 100, 200, and 300 loops were conducted to decrease the number of passes, which correspond to Figure 3b–d. The data shown in Figure 3(biii) proved that 10 passes were acceptable for complete cutting. Interestingly, the cutting lines are almost vertical in depth, which indicates a very small taper angle.

**Figure 4:**
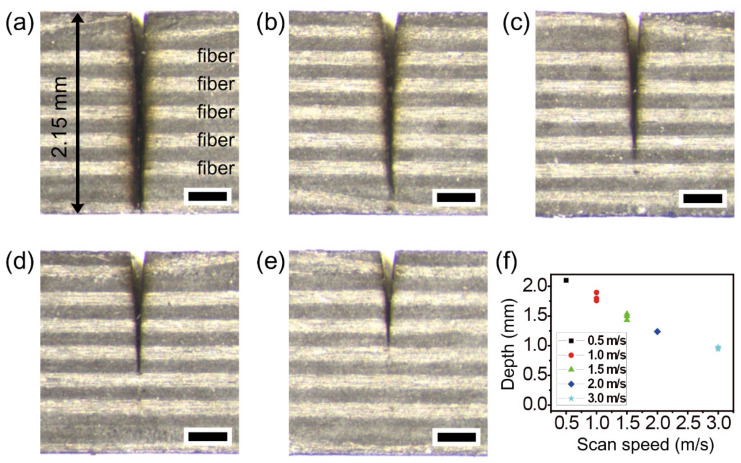
Picosecond laser cutting of the thermoset CFRP with scan speed variation. Stereoscopic microscope cross-section images with a scan speed of (**a**) 0.5, (**b**) 1.0, (**c**) 1.5, (**d**) 2.0, and (**e**) 3.0 m/s, respectively. Each scale bar = 400 μm. (**f**) Graph of cutting depths by scan speed variation. The common experimental conditions were a power of 22 W, 100 loops, and 10 passes with 10 μm gap.

Figure 4 shows that the scan speed is an important experimental parameter for deep cutting as other parameters are fixed. As seen in Figure 1b, the number of pulse irradiations per area is determined by scan speed. For this reason, a lower scan speed leads to deeper ablation in the depth direction. Figure 4a shows the complete cutting of 2.15 mm-thick thermoset CFRP at a scan speed of 0.5 m/s. The experimental results with scan speed variation at speeds of 1.0, 1.5, 2.0, and 3.0 m/s are shown in Figure 4b–e. With increasing scan speed, the HAZ areas slightly decreased, and the cutting depths also gradually decreased, as shown in Figure 4f. The common experimental conditions in Figure 4 were a power of 22 W, 100 loops, and 10 passes with a gap of 10 μm.

**Figure 5:**
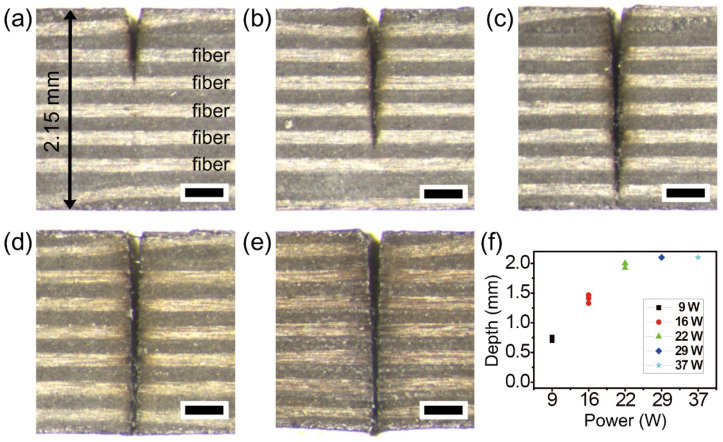
Picosecond laser cutting of the thermoset CFRP with power variation. Stereoscopic microscope cross-section images by a power of (**a**) 9, (**b**) 16, (**c**) 22, (**d**) 29, and (**e**) 37 W, respectively. Each scale bar = 400 μm. (**f**) Graph of cutting depths by power variation. The common experimental conditions were a scan speed of 0.5 m/s, 100 loops, and 10 passes with 10 μm gap.

To find the available maximum power for complete cutting of the thermoset CFRP, various powers were utilized up to 37 W. Figure 5 shows stereoscopic microscope cross-section images of (a) 9, (b) 16, (c) 22, (d) 29, and (e) 37 W, respectively. Increasing the power up to 22 W resulted in almost complete cutting with a slight heat effect, while the results with 29 and 37 W showed slight thermal damage near the cutting boundary. The effect on the cutting depth of power variation is shown in Figure 5b. The common experimental conditions in Figure 5 were a scan speed of 0.5 m/s, 100 loops, and 10 passes with a gap of 10 μm. The experimental results in Figure 5 indicate that a power of 22 W is acceptable for complete cutting with a negligible heat effect. Previous studies on the optimal laser fluence of maximal ablation efficiency reported F_0max_ ≈ 7.4 × F_th_ [38,39]. On the other hand, it is more or less difficult to generalize optimal fluence for maximal efficiency in CFRP materials because the material properties of the CFRPs are highly dependent on the polymer matrix.

**Figure 6:**
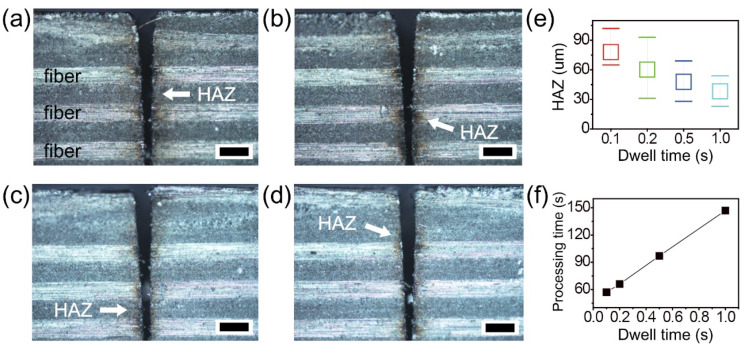
Investigation of heat-affected zone (HAZ) by picosecond laser cutting of the thermoset CFRP with dwell time variation. Optical microscope cross-section images with dwell time of (**a**) 0.1, (**b**) 0.2, (**c**) 0.5, and (**d**) 1.0 sec, respectively. The dwell time set after every 10 passes (here, one loop) in lateral direction. Each arrow indicates lateral HAZ near the cut surface. Each scale bar = 200 μm. Systematic investigation of (**e**) HAZ and (**f**) processing time by complete cutting as a function of dwell time. The common experimental conditions were a power of 22 W, a scan speed of 0.5 m/s, 100 loops, and 10 passes with 10 μm gap.

To achieve better cutting quality with a smaller HAZ, further experiments were carried out with dwell time variation (Figure 6). Optical microscope images of the top surface show HAZ areas in relation to dwell times of 0.1, 0.2, 0.5, and 1.0 s, which correspond to Figure 6a–d. The dwell times were set after every 10 passes (here, assumed as one loop) in the lateral direction. Each arrow in the images indicates the HAZ near the cutting area. A small HAZ of about 20 μm was measured, as shown in Figure 6d. Systematic investigation of HAZ and processing time was performed with 20 mm cutting length, and the results are summarized in Figure 6e,f. The average values of the HAZ were gradually decreased from 78, 60, 47, and 38 μm by increasing the dwell time (Figure 6e). Increased dwell time led to decreased HAZ, which caused longer processing time from 60 to 160 s (Figure 6f). The common experimental conditions in Figure 6 were a power of 22 W, a scan speed of 0.5 m/s, 100 loops, and 10 passes with a gap of 10 μm. Generally, a long machining time in ultrafast laser processing has been considered a major obstacle for industrial application. On the other hand, dwell time may not be a considerable issue and can be ignored, assuming that several machining tasks (e.g., 10 specimens) can be carried out at the same time.

**Figure 7:**
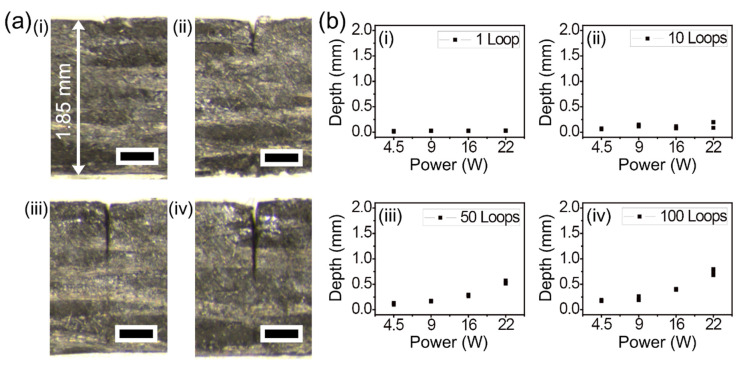
Picosecond laser cutting of the thermoplastic CFRP with lateral single-pass processing. (**a**) Stereoscopic microscope cross-section images by a power of 22 W and (i) 1 loop, (ii) 10 loops, (iii) 50 loops, and (iv) 100 loops, respectively. Each scale bar = 400 μm. (**b**) Systematic investigation of cutting depths by power variation and the number of loops variation with (i) 1, (ii) 10, (iii) 50, and (iv) 100. The common experimental condition was a scan speed of 0.5 m/s.

In an attempt to compare the machining characteristics of thermoplastic CFRP with thermoset CFRP, similar experimental parameters as those mentioned in previous paragraphs were employed for line cutting. Figure 7 shows the experimental results obtained by single-pass processing of 1.85 mm-thick thermoplastic CFRP by the same picosecond laser system. The stereoscopic microscope images show the cross-section of cutting in depth by (i) 1, (ii) 10, (iii) 50, and (iv) 100 loops, as shown in Figure 7a. To directly compare the results with the cutting results shown in Figure 2a, the same experimental conditions were utilized, namely a power of 22 W and a scan speed of 0.5 m/s. The cutting depth and kerf width of the thermoplastic CFRP were similar to the experimental results shown in Figure 2a. On the other hand, the thermoplastic CFRP was more sensitive to heat than the thermoset CFRP; therefore, high powers over 22 W could not be tested on account of thermal damage. Figure 7b displays the results obtained from systematic investigation of cutting depth using single-pass processing. The results with single-pass processing with power up to 22 W were confirmed to be very similar to those shown in Figure 2b. Additionally, all the cases seemed to be unable to cut completely, which was also true for the thermoset CFRP.

**Figure 8:**
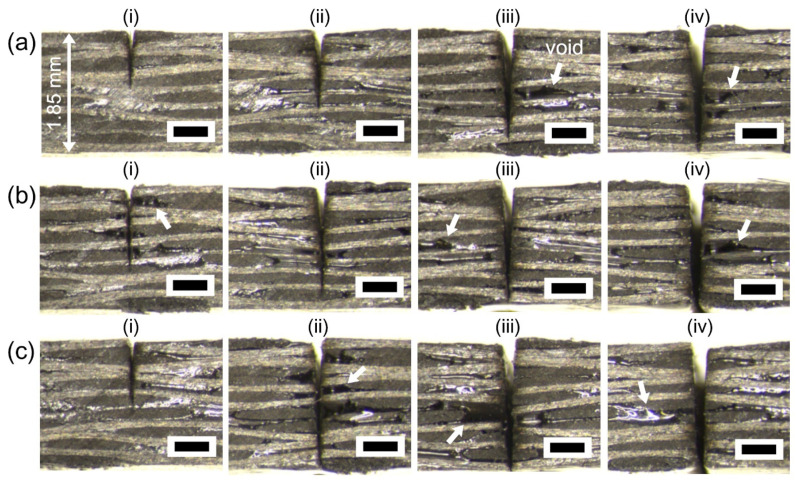
Picosecond laser cutting of the thermoplastic CFRP with lateral multi-pass processing. Stereoscopic microscope cross-section images with (**a**) 50 loops, (**b**) 100 loops, and (**c**) 200 loops, respectively. Each position at (i), (ii), (iii), and (iv) corresponds to multi-pass processing of 5, 10, 15, and 20 passes in lateral direction with 10 μm gap. The common experimental conditions were a power of 16 W and a scan speed of 0.5 m/s. Each scale bar = 500 μm.

To elucidate the effect of multi-pass processing, the number of passes was systematically investigated with 5, 10, 15, and 20 lateral passes with a gap of 10 μm, which correspond to (i), (ii), (iii), and (iv) in Figure 8. The common experimental conditions were a power of 16 W and a scan speed of 0.5 m/s. According to the equation regarding the diameter squared versus fluence method, a spot size is 24.9 µm has a fluence of 6.37 J/cm^2^ (800 kHz, 16 W). A stereoscopic microscope image in Figure 8a verifies that 50 loops are acceptable for complete cutting when 20 passes are used. Additional works with 100 and 200 loops were carried out to decrease the number of passes, which correspond to Figure 8b,c. The data shown in Figure 8(ciii) demonstrate that 15 passes were capable of complete cutting. With a proper kerf width for multi-pass processing, complete cutting of thermoplastic CFRP was achieved, but thermal damages were observed (marked with arrows in Figure 8). These results suggest that thermoplastic CFRP is vulnerable to heat damage despite the use of a green picosecond laser, and it is more sensitive to thermal damage than the thermoset CFRP.

**Figure 9:**
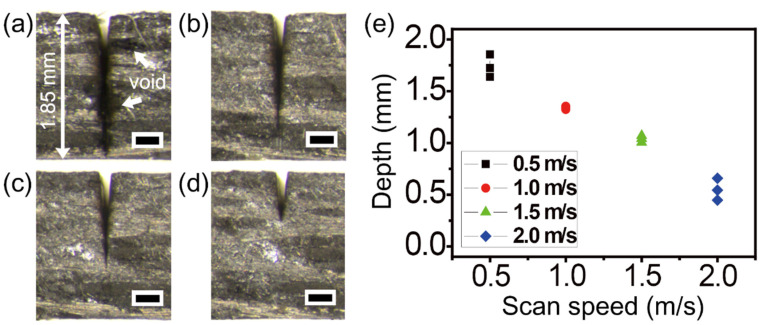
Picosecond laser cutting of the thermoplastic CFRP with different scan speeds. Stereoscopic microscope cross-section images with scan speed of (**a**) 0.5, (**b**) 1.0, (**c**) 1.5, and (**d**) 2.0 m/s, respectively. Each scale bar = 300 μm. (**e**) Graph of cutting depths by scan speed variation. The common experimental conditions were a power of 22 W, 100 loops, and 10 passes with 10 μm gap.

To avoid heat accumulation-induced thermal damage of thermoplastic CFRP, faster scan speeds were investigated. Figure 9 shows experimental results obtained with scan speed variation to speeds of 0.5, 1.0, 1.5, and 2.0 m/s, which correspond to Figure 9a–d. Although the scan speed of 0.5 m/s led to complete cutting, several voids in the HAZ were observed near the cutting area (Figure 9a). In contrast, with increased scan speed, the HAZ areas were dramatically decreased. As seen in Figure 1b, the effective shot numbers (the number of pulse irradiations per area) are determined by scan speed. For this reason, a faster scan speed caused decreased effective shot numbers; hence, the cutting depth was considerably decreased, as shown in Figure 9d. The relationship between the scan speed and machining depth is shown in Figure 9e. The common experimental conditions in Figure 9 were a power of 22 W, 100 loops, and 10 passes with a gap of 10 μm.

This study was performed to directly compare the experimental parameters for thermoset and thermoplastic CFRP machining using a 1 ps green laser. The results regarding the thermoset CFRP show that it is possible to use a longer pulse of up to 10 ps for the purpose of increasing ablation efficiency [40]. On the other hand, the thermoplastic CFRP might require a sub-pico or femtosecond laser with low repetition rate to be used, in order to avoid thermal damage caused by heat accumulation. Although a nanosecond laser is known as one of the good options for laser micromachining, it is not suitable for CFRP machining due to causing thermal damage. The choice of laser wavelength could influence the ablation efficiency and therefore, the characteristics of the polymer matrix should be considered. In particular, an appropriate laser wavelength can lead to better cutting quality of thermoplastic CFRP. Therefore, further studies on the laser machining of thermoplastic CFRP with various polymer matrices are necessary.

**Figure 10:**
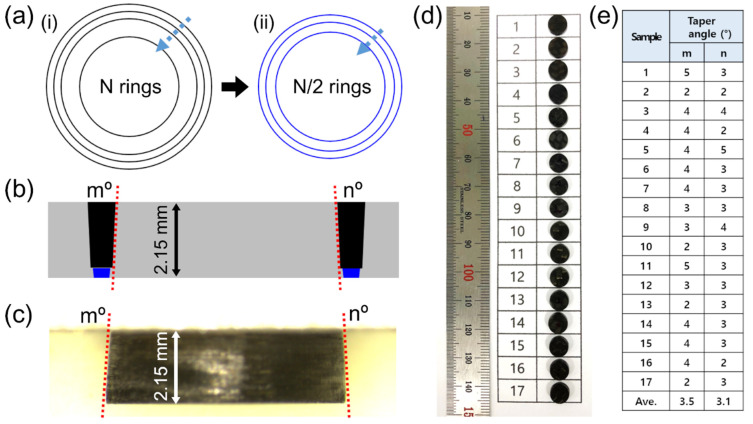
Taper angles of the drilled cylinder specimens of the thermoset CFRP. (**a**) Schematic of hole drilling strategy by multi-ring scanning with two-step processing. (**b**) Schematic of cross-section for the two steps of multi-ring scanning. Taper angles on left and right side correspond to m° and n°, respectively. (**c**) Stereoscopic microscope cross-section image of the laser-drilled cylinder specimen. (**d**) The photograph of 17 specimens used for investigation of taper angle. (**e**) Systematic investigation of the taper angles of the specimens as shown in (**d**). Average values are between 3° and 3.5°.

With reference to the machining parameters of line cutting discussed previously, we delved into the experimental parameters for hole drilling. Figure 10a presents a schematic of the multi-step strategy for picosecond laser hole drilling. The purpose of the first step with low power and multi-ring processing is to make a wide kerf width and to avoid thermal effects. In the subsequent step, half as many multi-rings as those used in the first step with higher power were used to penetrate the residual layers of the specimen. This two-step strategy achieved efficient machining with a shorter processing time to drill 2.15 mm-thick CFRP samples. Figure 10b shows a schematic of cross-sections obtained by hole drilling with two-step processing. The expected taper angles by Gaussian beam processing are indicated by red lines and characters m° and n°, respectively. Interestingly, the taper angles of the 2.15 mm-thick specimens were quite small, as shown in the stereoscopic microscope cross-section image in Figure 10c. A lot of drilling tasks were carried out by two-step processing, and photographs of the drilled samples are displayed in Figure 10d. The taper angles of all the specimens shown in Figure 10d were measured by a stereoscopic microscope, and the results are summarized in Figure 10e. The average values were between 3° and 3.5° for m and n, respectively. The common experimental conditions utilized for the first step were 15 to 20 rings, a scan speed of 0.5 m/s, a power of 22 to 29 W, and 100 loops. The second step was performed with 7 to 10 rings, a scan speed of 0.5 m/s, a power of 29 to 37 W, and 100 loops. The multi-pass in lateral direction using the small beam spot size (e.g., 25 μm) versus kerf width (e.g., 200 μm) had effects on the decrease in taper angle, compared to use of a large beam spot size. In addition, the higher power used in the second step may have a slight influence to make a wider beam exit when it penetrated the residual layer near bottom.

**Figure 11:**
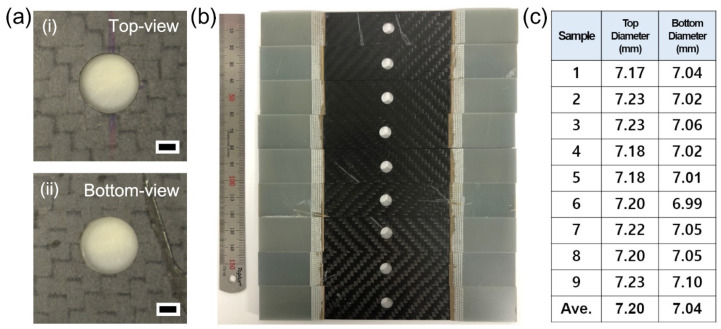
Laser-drilled hole diameters of the thermoset CFRP. (**a**) Stereoscopic microscope images of laser-drilled hole with (i) top view and (ii) bottom view. Each scale bar = 2 mm. (**b**) Photograph of laser-drilled specimens for tensile strength tests. (**c**) Systematic investigation of hole diameters of top and bottom sides as shown in (**b**). Average values of the diameters are 7.2 and 7.04 mm for the top and bottom sides.

To further investigate the small taper angles of the specimens shown in Figure 10, the hole diameters at the top (beam entrance) and bottom side (beam exit) were examined, as shown in Figure 11. Figure 11a displays the top and bottom views of a drilled specimen, which seems to show less heat effects in the low-magnification image. A variety of drilled specimens were prepared for tensile strength tests, as shown in Figure 11b. All of the hole diameters of both the top and bottom side were measured, and the results are summarized in Figure 11c. The average values were 7.20 mm for the top side and 7.04 mm for the bottom side. The similar hole diameters of the top and bottom side coincide well with the measured small taper angles shown in Figure 10e. This result regarding small taper angle is very important for application to the SPR for dissimilar CFRP/metal joints. In an effort to reduce the taper angle, precision machining was developed and the effect of the top-hat beam was investigated [41,42]. Those methods might be not suitable for millimeter-scale machining of CFRP materials due to thermal effects and low processing time. On the other hand, our method demonstrated the feasibility of millimeter-scale machining of CFRP materials.

**Figure 12:**
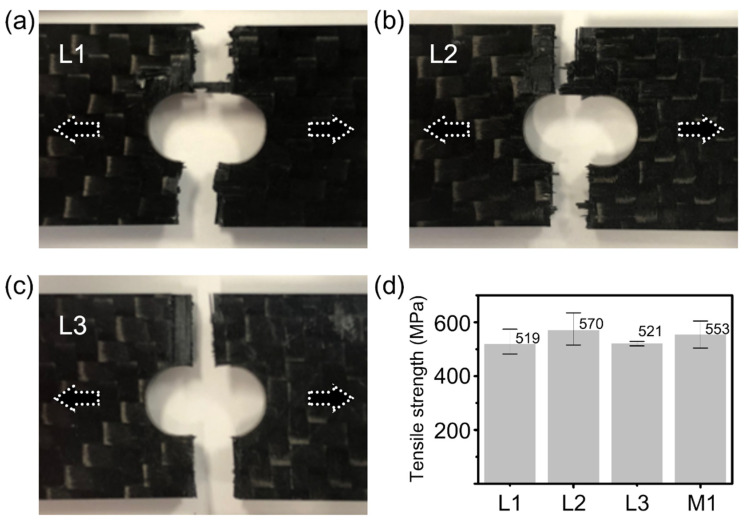
Tensile strength tests of 2.15 mm thickness specimens. The results of tensile strength tests for the specimens, prepared by three different experimental conditions, correspond to (**a**) L1, (**b**) L2, and (**c**) L3, respectively. Each hole diameter = 7 mm. (**d**) Comparison data of tensile strength for picosecond laser processing (L1, L2, and L3) and mechanical drilling processing (M1).

Figure 12 shows the comparison results of tensile strength testing of the 2.15 mm-thick specimens subjected to the picosecond laser and mechanical drilling. For the tensile strength test, thermoset CFRP plates of 150 × 15 mm were prepared by water jet cutting, and three different experimental conditions (L1, L2, and L3) for the laser drilling process were performed in the middle of the CFRP plates. The experimental conditions used for L1 were a power of 22 W, 20 passes, and 200 loops for the first step and a power of 37 W, 10 passes, and 50 loops for the second step. The common conditions were a scan speed of 0.5 m/s and dwell time of 1 s per 5 passes. The following conditions for L2 were employed with a dwell time of 0.5 s per 5 passes to reduce total processing time, and other conditions were the same as those of L1. The last conditions for L3 were tested using a power of 37 W for both the first and the second step, in order to decrease the number of loops. The optimized conditions used for L3 were 100 loops for each step. Furthermore, mechanically drilled samples (M1) of the highest quality were also prepared to compare with the laser-drilled specimens. All specimens were tested according to KS M ISO 527-4; the results of laser-drilled specimens are displayed in Figure 12a–d and are compared to the tensile strength test results obtained for the specimens produced by laser drilling and mechanical drilling. Average values for L1, L2, L3, and M1 correspond to 519, 570, 521, and 553 MPa. Although the L3 using a higher power was expected to have a little damage, the tensile strength results were shown to be similar to those of L1. The best result was obtained by the L2 specimens.

## 4. Conclusions

In summary, green picosecond laser micromachining of both 2.15 mm-thick thermoset and 1.85 mm-thick thermoplastic CFRPs was investigated for application in self-piercing riveting (SPR) of CFRP/metal dissimilar joining in the automotive industry. A variety of experimental parameters were systematically examined and optimized to produce a smaller HAZ and resulted in complete cutting of thermoset and thermoplastic CFRPs. Stereoscopic microscopy was conducted to verify the millimeter-scale thickness and taper angles of the samples at once from top to bottom. Our experimental results identified that the thermoplastic CFRP was more sensitive to heat and required a longer processing time with a lower power and faster scan speed in comparison to the thermoset CFRP. With reference to the machining parameters obtained from line cutting, multi-ring-based hole drilling was performed. Both the taper angles and hole diameters of the top and bottom-side of all the drilled specimens were evaluated, resulting in average taper angles between 3° and 3.5°. This small taper angle is crucial for the SPR application. In addition, tensile strength tests were carried out to characterize the mechanical property of the laser-drilled specimens (570 MPa), and the results were compared with those obtained for mechanically drilled specimens. This work is expected to provide potential opportunities for application in green picosecond laser micromachining of CFRP materials with a small taper angle and narrow heat-affected zone.

## Figures and Tables

**Table 1:** Specifications of green picosecond laser system.

Parameters	Specifications
Operating mode	Pulsed
Wavelength	515 nm
Max. average power	200 W
Repetition rate	800 kHz
Pulse length	1 ps
Field of view of f-theta lens	140 mm
Focal length	170 mm
Max. galvanometer scanning speed	6 m/s
Beam waist diameter	20 μm (1/e^2^)
